# A Case of Adult-Onset Eccrine Angiomatous Hamartoma—The Comparison with Epithelioid Hemangioma

**DOI:** 10.3390/dermatopathology9020012

**Published:** 2022-03-25

**Authors:** Mai Nishimura, Yoshiaki Matsushima, Yasuo Nakai, Koji Habe, Akinobu Hayashi, Keiichi Yamanaka

**Affiliations:** 1 Department of Dermatology, Graduate School of Medicine, Mie University, 2-174 Edobashi, Tsu 514-8507, Mie, Japan; ishika-m@med.mie-u.ac.jp (M.N.); matsushima-y@clin.medic.mie-u.ac.jp (Y.M.); nakai-y@clin.medic.mie-u.ac.jp (Y.N.); habe-k@clin.medic.mie-u.ac.jp (K.H.); 2 Department of Oncologic Pathology, Graduate School of Medicine, Mie University, 2-174 Edobashi, Tsu 514-8507, Mie, Japan; ahayashi@doc.medic.mie-u.ac.jp

**Keywords:** eccrine angiomatous hamartoma (EAH), epithelioid hemangioma (EH), hyperplasia of normal or dilated eccrine glands, S100, anti-EMA

## Abstract

Eccrine angiomatous hamartoma (EAH) is a relatively rare benign skin disease characterized by the proliferation of eccrine sweat glands associated with capillary hemangioma and the proliferation of other skin elements such as adipose tissue, hair, and epidermis. The onset of the disease is usually at birth or in childhood and tends to occur in the extremities of females, but it occurred in an adult male in this case. The patient was a 72-year-old man with a 12 × 12 mm light brown, elastic, slightly firm skin nodule on the flexor aspect of his right forearm. A biopsy revealed enlargement of blood vessels, sweat glands, sweat ducts, and erector spongiosum with both lumen dilation and narrowing, leading to the diagnosis of EAH. The histopathological features of EAH include a marked proliferation of microvessels, epithelial-like changes in vascular endothelial cells (such as enlarged nuclei), and infiltration of inflammatory cells, mainly lymphocytes and plasma cells. In adult-onset cases, EAH can be clinically difficult to distinguish from epithelioid hemangioma (EH), which differs in the predominance of microvascular proliferation and the presence of eosinophils in the infiltrating inflammatory cells. It can also be distinguished from EAH by the negative results of S100 and anti-EMA in immunohistological staining. In the current cases, we were able to differentiate the two cases from characteristic findings on HE staining.

## 1. Introduction

Eccrine angiomatous hamartoma (EAH) is a relatively rare benign skin disease first described by Lotzbeck [1] in 1859 as a lesion of hemangiomatous tissue with a proliferation of eccrine sweat glands, and defined as EAH by Hyman et al. [2]. EAH is a solitary erythematous nodule with a predilection for infancy and childhood. It usually occurs on the hands and feet but can also appear on other parts of the body. In recent years, some cases have been reported in adults, and it is sometimes difficult to clinically distinguish EAH from epithelioid hemangioma (EH). In the present study, we report a case of EAH in an adult based on clinical and histopathological examinations and compare it with a case of EH.

## 2. Case Report

### 2.1. Case 1

A 72-year-old man noticed a skin rash on his right forearm while working in his garden two months prior to presentation. Assuming it was a bug bite, he ignored it, but it did not improve. He decided that it was a thorn prick and punctured it himself, but no thorn was found. One month later, he visited a local dermatologist who diagnosed the rash as a local infection with desquamation and exudate on the surface; he was prescribed minocycline 100 mg/day for 12 days. Since there was no improvement after this and tenderness was observed, the patient was referred to our department.

**Figure 1:**
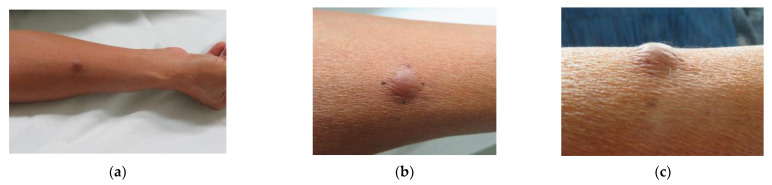
Clinical picture: (**a**) a 12 × 12 mm pale brown, elastic, slightly firm, dermal nodule on the flexor aspect of the right forearm; (**b**) Front view; (**c**) Side view.

**Figure 2:**
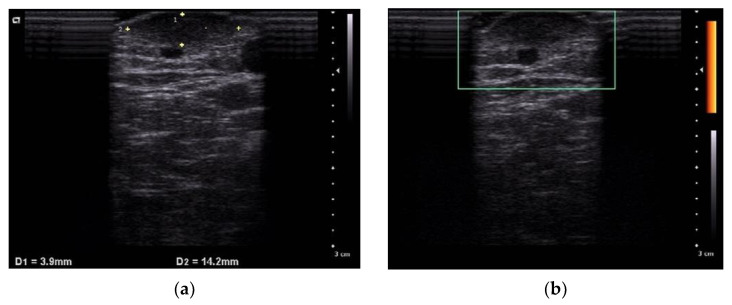
Echo ultrasonography shows a slightly hypoabsorbent nodule with indistinct borders (**a**). Doppler image; No internal blood flow is observed (**b**).

At the time of initial examination, there was a 12 × 12 mm pale brown, elastic, slightly firm, dermal nodule on the flexor aspect of the right forearm that was adherent to the epidermis but had good mobility with the lower floor (Figure 1a–c). Although slight tenderness was noted, there was no hyperhidrosis or hypertrichosis. Echo ultrasonography showed a slightly hypoabsorbent nodule with indistinct borders and no internal blood flow (Figure 2a,b).

**Figure 3:**
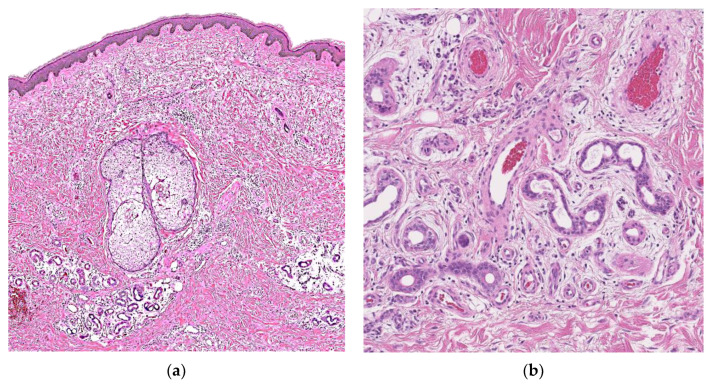
Hematoxylin and eosin staining shows increased vascularity, sweat glands and sweat ducts with lumen dilation and narrowing. There were numerous capillary channels surrounding or intermingled with the eccrine structures: (**a**) ×100 magnification; (**b**) ×400 magnification.

Excisional biopsy revealed increased vascularity, sweat glands, and sweat ducts with lumen dilation and narrowing (Figure 3a,b). There were numerous capillary channels surrounding or intermingled with the eccrine structures. Fibroblast proliferation was observed around the sweat glands, accompanied by edema. Large malformed sebaceous glands without continuity with hair follicles were observed. Although the hemangioma component was scant, we diagnosed EAH because of its relationship to eccrine glands. He has passed without any recurrence. 

### 2.2. Case 2

**Figure 4:**
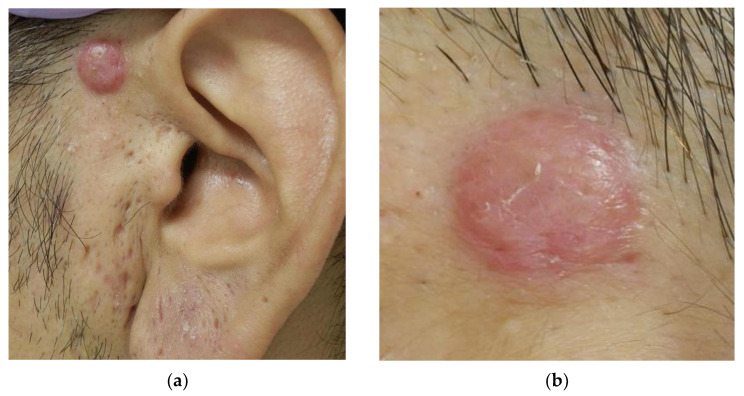
(**a**) A 10 × 11 mm red dome-shaped nodule above the anterior part of the left ear; (**b**) Enlarged image.

**Figure 5:**
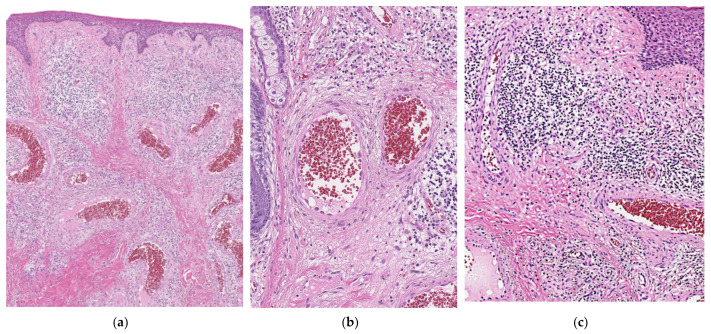
Dilated blood vessels in all layers of the dermis and enlarged endothelial cells lining blood vessels are observed: (**a**) ×200 magnification; (**b**) ×400 magnification; (**c**) Inflammatory cell infiltration with few eosinophils is observed in the surrounding area (×400 magnification).

A 40-year-old man noticed a 10 × 11 mm red dome-shaped nodule above the anterior part of the left ear for over a year (Figure 4a,b). There were no subjective symptoms, and excisional biopsy revealed dilated blood vessels in all layers of the dermis and enlarged endothelial cells lining blood vessels (Figure 5a,b). In addition, inflammatory cell infiltration with few eosinophils was observed in the surrounding area (Figure 5c), which led to the diagnosis of EH. After the resection, the patient passed without any problems.

## 3. Discussion

It is known that the majority of EAH cases develop at birth or in childhood as a hypermetropic disorder characterized by the proliferation of eccrine sweat glands associated with capillary hemangiomas and the proliferation of other skin elements such as adipose tissue, hair, and epidermis; more common in women, and the most typical site of occurrence is in the extremities. In recent years, there have been a few reports of adult-onset cases, some of which have been triggered by external stimulus [3,4,5,6]. In this case, it could have been induced by an insect bite or by skin puncture. The clinical features of EAH include nodules, masses, and patches that vary in color from brown to red, purple-red, and normal skin color. Local tenderness and hyperhidrosis may or may not be present. Histopathological evaluation is necessary for the definitive diagnosis of EAH, and, in most cases, the lesions are located in the dermis. Pelle et al. defined the diagnostic criteria for EAH and included hyperplasia of normal or dilated eccrine glands; close association of eccrine structures with capillary hemangioma structures; and the presence of hair, adipose tissue, mucous components, and lymphatic vascular structures in their criteria [7]. This case does not fulfil all the requirements/underpinnings of classic eccrine angiomatous hamartoma rather than the criteria of a “forme fruste”-like variant of EAH, although so far for these lesions, no clearly defined histopathological terminology exists. Immunohistochemical (IHC) staining for EAH shows similar findings to normal eccrine structures: Secretory portions are positive S100 protein for eccrine structures, CAM5.2 for epithelial cells, anti-epithelial membrane antigen (EMA) for glandular epitheliums, and anti-carcinoembryonic antigen (CEA) for eccrine structures. Tubular portions are positive anti-CEA and anti-EMA [8]. Factor VIII-associated antigen and anti-Ulex Europaeus Lection 1 antibody are useful for identifying vascular components [9].

Generally, EH is a benign disease known to form reddish-brown nodules with a predilection for the skin or subcutaneous tissues of the head, neck, and extremities. Histopathological features of EH include a marked proliferation of microvessels, enlarged nuclei of vascular endothelial cells, epithelial-like changes, and occasional vacuolation of nuclei [10]. The stroma is characterized by inflammatory cell infiltration, mainly by lymphocytes and plasma cells, and often by eosinophils. IHC staining is negative for S100 and anti-EMA, unlike EAH [11].

From the above, the lesions of EH are mainly vascular proliferation and can be distinguished from EAH by the absence of eccrine sweat gland-like structures and eosinophils. If HE staining is difficult to diagnose, IHC staining for S100 and anti-EMA is helpful. In the present case, both showed typical findings on HE staining and could be diagnosed.

Clinically, the other differential diagnosis of EAH includes eccrine nevus, tufted angioma, smooth muscle hamartoma, and blue rubber bleb nevus syndrome (Table 1). The histopathologic examination aids in the distinction from these entities, but eccrine nevus may display histologic resemblance. The absence of angiomatous hyperplasia in eccrine nevus, a rare entity characterized by groupings of normal to enlarged eccrine structures, distinguishes it from EAH.

## 4. Conclusions

We examined EAH that developed in an adult male. In the current case, the hemangioma component was scant, but the presence of capillaries closely associated with proliferating eccrine glands allowed the diagnosis of EAH. In adult cases, it is difficult to distinguish EAH from EH, and the presence of eccrine glands is important. If a diagnosis is difficult with HE staining, IHC staining for S100 and anti-EMA may be helpful.

## Figures and Tables

**Table 1:** EAH and the clinical differential diagnoses of EAH, including the features of demography and histopathological findings.

Disease	Number of Cases Reported in English	Histopathological Finding	Reference
EAH	<100	the lesion affects mainly mid and deep dermis and is composed of lobules of proliferating capillaries intricately admixed with sweat glands and ducts, fat and myxoid tissue.	[12]
Eccrine nevus	20	It is characterized by groupings of normal to enlarged eccrine structures. (The absence of angiomatous hyperplasia)	[13]
Tufted angioma	200	multiple, scattered lobules of small capillary type vessels with small oval to spindle shaped cells throughout the dermis and subcutaneous tissue imparting a “cannonball” or glomerular appearance.	[14]
Smooth muscle hamartoma	<20 (Only acquired type)	It shows disseminated proliferation of mature smooth muscle cells of a central cigar-shaped nucleus and fibrillary and eosinophilic cytoplasm.	[15,16]
Blue rubber bleb nevus syndrome	200	cutaneous lesions are non-specific and have features of venous malformations. Large, tortuous, dilated vessels with a single endothelial lining are noted, and smooth muscle may be present in the vessel walls.	[17]

## Data Availability

The patients’ data are not publicly available on legal or ethical grounds.

## References

[1] Lotzbeck C. (1859). A case of sweat gland tumor on cheek. Virchows Arch..

[2] Hyman A.B., Harris H., Brownstein M.H. (1968). Eccrine angiomatous hamartoma. N. Y. State J. Med..

[3] Yoshida M.N.T., Kawata A. (2013). A case of eccrine angiomatous hamartoma. JJCD.

[4] Naik V., Arsenovic N., Reed M. (2009). Eccrine angiomatous hamartoma: A rare multifocal variant with features suggesting trauma. Dermatol. Online J..

[5] Hawryluk E.B., Schmidt B., Maguiness S. (2015). Enlargement of eccrine angiomatous hamartoma following trauma. Pediatr. Dermatol..

[6] Gadroy A., Belhadjali H., Bayle P., Albes B., Lamant L., Bazex J. (2003). Eccrine angiomatous hamartoma: An atypical case. Ann. Dermatol. Venereol..

[7] Pelle M.T., Pride H.B., Tyler W.B. (2002). Eccrine angiomatous hamartoma. J. Am. Acad. Dermatol..

[8] Cebreiro C., Sanchez-Aguilar D., Gomez Centeno P., Fernandez-Redondo V., Toribio J. (1998). Eccrine angiomatous hamartoma: Report of seven cases. Clin. Exp. Dermatol..

[9] Garcia-Arpa M., Rodriguez-Vazquez M., Cortina-de la Calle P., Romero-Aguilera G., Lopez-Perez R. (2005). Multiple and familial eccrine angiomatous hamartoma. Acta Derm. Venereol..

[10] Fetsch J.F., Weiss S.W. (1991). Observations concerning the pathogenesis of epithelioid hemangioma (angiolymphoid hyperplasia). Mod. Pathol..

[11] Sun Z.J., Zhang L., Zhang W.F., Alsharif M.J., Chen X.M., Zhao Y.F. (2006). Epithelioid hemangioma in the oral mucosa: A clinicopathological study of seven cases and review of the literature. Oral. Oncol..

[12] Apte A., Nema P., Bandi A. (2017). Eccrine angiokeratomatous hamartoma: Case report of a 1.5-year girl. J. Surg. Case Rep..

[13] Chien J.A., Asgari M., Argenyi B.Z. (2006). Eccrine angiomatous hamartoma with elements of an arteriovenous malformation: A newly recognized variant. J. Cutan. Pathol..

[14] Tjarks J.S.C.S. Tufted Tumors. https://www.pathologyoutlines.com/topic/skintumornonmelanocyticacquiredangioma.html.

[15] Raboudi A.L.N. Congenital Smooth Muscle Hamartoma. https://www.ncbi.nlm.nih.gov/books/NBK545188/.

[16] Ladha A.M., Remington T. (2019). Acquired smooth muscle hamartoma: A case report on the lower extremity hidrosis. SAGE Open Med. Case Rep..

[17] Baigrie D.R.S.A. Blue Bubber Bleb Nevus Syndrome. https://www.ncbi.nlm.nih.gov/books/NBK541085/.

